# Influence of the roughness of dental implants obtained by additive manufacturing on osteoblastic adhesion and proliferation: A systematic review

**DOI:** 10.1016/j.heliyon.2022.e12505

**Published:** 2022-12-25

**Authors:** Juliana Dias Corpa Tardelli, Ana Carolina Duarte Firmino, Izabela Ferreira, Andréa Cândido dos Reis

**Affiliations:** Department of Dental Materials and Prosthesis, School of Dentistry of Ribeirão Preto, University of São Paulo (USP), Ribeirão Preto, SP, Brazil

**Keywords:** Additive manufacturing, Dental implant, Roughness, Osteoblast, Cell viability, Cell adhesion

## Abstract

**Objective:**

Critically analyzed the existing literature to answer the question "What is the influence of roughness of surfaces for dental implants obtained by additive manufacturing compared to machined on osteoblastic cell adhesion and proliferation?"

**Design:**

This systematic review followed the guidelines of the Preferred Reporting Items for Systematic Reviews and Meta-Analyses (PRISMA) and was registered in the Open Science Framework. The personalized search strategy was applied to Embase, Pub Med, Scopus, and Science Direct databases and Google Scholar and ProQuest grey literature. The selection process was carried out in two stages independently by two reviewers according to the eligibility criteria. The risk of bias was analyzed using a checklist of important parameters to be considered.

**Results:**

When applying the search strategy on databases 223 articles were found, after removing the duplicates, 171 were analyzed by title and abstract of which 25 were selected for full reading, of these, 6 met the eligibility criteria. 2 studies were included from the reference list totaling 8 articles included in this systematic review and none were included from the Grey Literature. 7 had a low risk of bias and 1 moderate.

**Conclusions:**

1) Roughness is a property that must be analyzed and correlated with the chemical composition, intrinsic to the alloy and resulting from the surface treatment; morphology of topographic peaks and valleys; printing technique and its parameters; 2) Need for more studies on the biomolecular level to elucidate the mechanism by which the roughness and the morphology of topographical peaks and valleys descriptive of roughness influence osteoblastic adhesion and proliferation.

## 1 Introduction

Additive manufacturing is the most promising technique for the production of porous implants because it allows control of the size, morphology, and distribution of pores [1, 2, 3, 4, 5, 6, 7, 8, 9, 10]. In addition to being different from conventional techniques, machining, and casting, it reduces the time and material used and allows the production of customized complex structures eliminating the need for invasive tissue manipulation [1, 2, 3, 4, 5, 6, 7, 8, 9, 10]. There are several techniques currently available, such as direct laser fabrication (DLF), electron beam melting (EBM), and selective laser melting (SLM). In some techniques, the porosity and mechanical strength of the structure can be controlled by adjusting parameters such as the orientation angle, layer height, melting temperature, printing speed, powder particle morphology, and energy flow [11, 12, 13, 14].

However, additional processing is required on porous surfaces due to unfused surface metallic residues that can cause peri-implant inflammation and affect rehabilitation survival [1, 4, 15, 16, 17, 18]. Among them, polishing minimizes fatigue failures by stress concentration in irregular areas and can be associated with physical, chemical, and biological methods [5, 9, 19]. Particular attention should be given to surface treatments since they change not only the roughness, but also the composition chemistry directly impacting cytocompatibility and other physical, chemical, and biological properties [19, 20].

The Ti–6Al–4V alloy is the most used for biomedical applications due to its biocompatibility, high strength/weight ratio, corrosion resistance, and bioinert oxide surface concerning cobalt and stainless steel alloys [7, 8, 10, 21, 22]. However, its modulus of elasticity is incompatible with that of bone tissue, which may lead to aseptic loosening of the implant due to the phenomenon of protection against tensions, in which the implant, being more rigid than the bone tissue, supports most of the load and the bone is reabsorbed by lack of stimuli following Wolff's law [21, 23, 24, 25, 26].

To overcome this incompatibility, two strategies are proposed for the production of implants of beta titanium alloys, which have a lower elastic modulus, and porous implants, produced by additive manufacturing, in which their porous mesh can be customized for the desired rigidity, in addition to providing greater surface area for adhesion, fixation and osteoblast proliferation [7, 8, 10, 21, 22, 27, 28].

Contact osteogenesis is influenced by surface characteristics, topography, hydrophilicity, and roughness, the latter in printed implants is Ra around 25 μm [29, 30, 31, 32]. According to the literature, Ra surfaces of 3–5 μm are ideal for the osteoblastic response [5, 33, 34], however, there is still no consensus on the influence of surfaces above Ra 5 μm on osteoblastic activity, only that they favor mechanical imbrication and mimic the hierarchical structure of bone tissue [5, 29, 32, 35]. Therefore, the objective of the present systematic review was to evaluate the existing literature to answer the question "What is the influence of roughness of surfaces for dental implants obtained by additive manufacturing compared to machined on osteoblastic cell adhesion and proliferation?".

## 2 Material and methods

### 2.1 Protocol

This systematic review was prepared according to the Preferred Reporting Items for Systematic Review and Meta Analyzes Protocols (PRISMA 2020) [36] and registered in the Open Science Framework (osf.io/sb9my) to answer the question "What is the influence of roughness of surfaces for dental implants obtained by additive manufacturing compared to machined on osteoblastic cell adhesion and proliferation?". The acronym PECOS contemplated in this systematic review was: Population = surfaces for dental implants obtained by additive manufacturing; Exposure = roughness; Comparison = surfaces for dental implants obtained by machining; Outcome = adhesion or proliferation of osteoblasts, Studies = experimental *in vitro* studies.

### 2.2 Eligibility criteria

*In vitro* studies comparing the effect of roughness of implants obtained by additive manufacturing versus machining on osteoblastic proliferation and adhesion were included, without a time and language restriction. And excluded: 1) Did not evaluate surfaces obtained by additive manufacturing; 2) Did not evaluate surfaces obtained by machining; 3) Bone plates; 4) Compared machined surface with those obtained by additive manufacturing treated superficially; 5) Scaffold; 6) Did not mention additive manufacturing technique; 7) Orthopedic implant.

### 2.3 Search strategy

The personalized search strategy was applied to the Embase, PubMed, Scopus, and Science Direct databases and Google Scholar and ProQuest grey literature on October 03rd, 2022, without the restriction of time (Appendix 1). The EndNote X8 software was used to remove duplicates and the Rayyan web application was used to select articles by title and abstract.

### 2.4 Selection process

Reviewers J.D.C.T and A.C.D.F independently evaluated the articles found in two phases according to the eligibility criteria. The first phase involved selecting based on the title and abstract, and the second phase involved reading the entire article. Doubts and discrepancies were resolved at the consensus meeting with the third reviewer I.F and coordinator A.C.R.

### 2.5 Data tabulation

J.D.C.T and A.C.D.F tabulated data in a Word spreadsheet independently according to author, year; groups; additive manufacturing technique; method to assess roughness; roughness results; method to assess adhesion, and/or osteoblastic proliferation; results; expressed in Appendix 2.

### 2.6 Risk of bias analysis

The risk of bias in the studies was analyzed as previously performed by Sarkis-Onofre et al., 2014 [37] according to the description of important parameters to be analyzed: clarity in the materials section, presence of suitable groups for comparison, clarity in the methodology section, roughness, and osteoblastic adhesion/proliferation assessed in a reliable method, sufficient detail to allow replication, clarity of results. As for the parameters reported, the article is scored with "Y" (yes), if not "N" (no). The classification of risk of bias was performed according to the number of parameters reported, as of 6 or 5 items low risk of bias, 4 or 3 moderate risks of bias, and 2 or 1 high risk of bias. Graphical analysis was performed using RevMan 5.3 software.

## 3 Results

### 3.1 Article selection process

**Figure 1:**
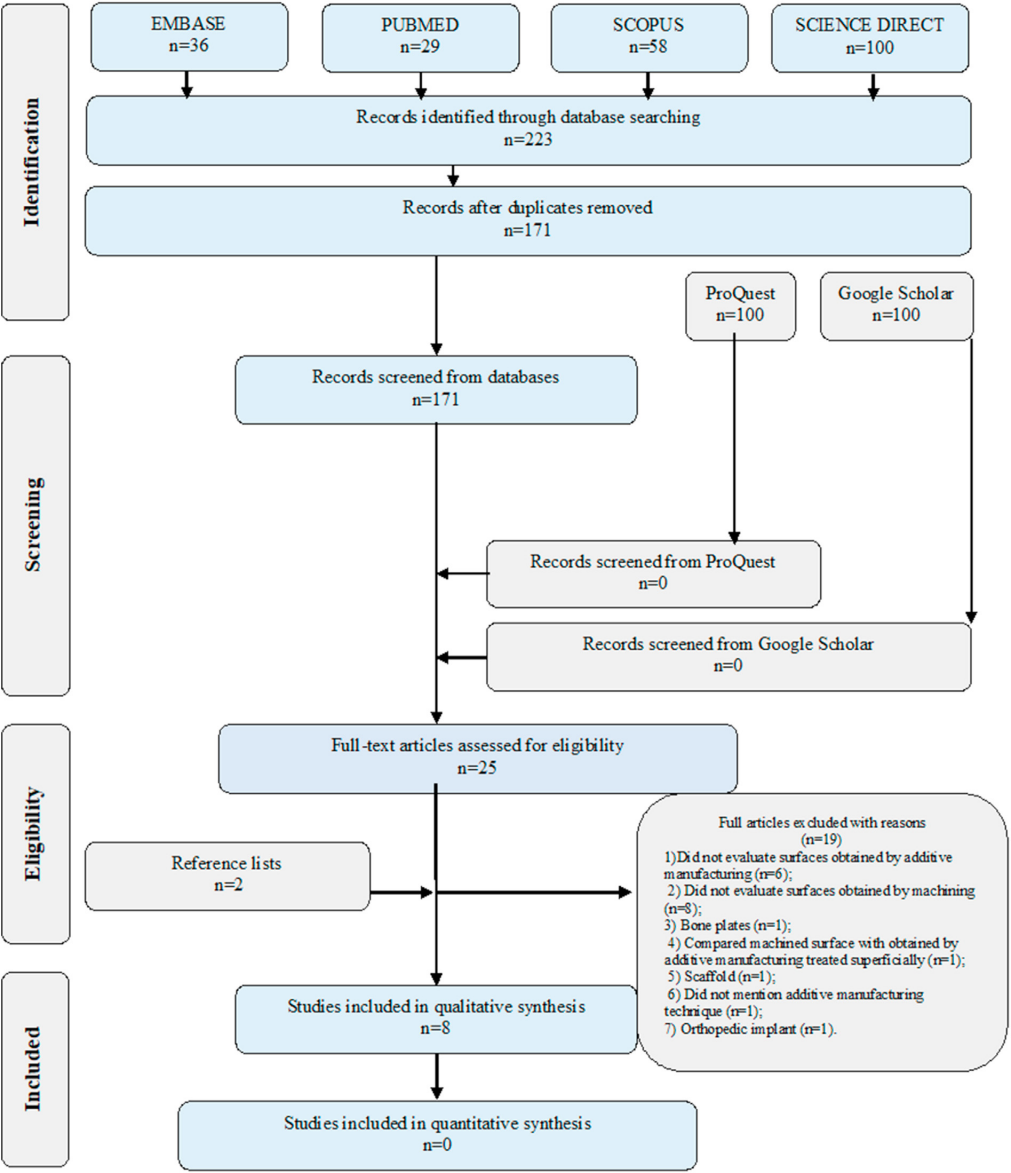
Flow diagram of literature search and selection criteria.

After the application of the personalized search strategy in the Embase, PubMed, Scopus, and Science Direct databases, 223 articles were found, after removing the duplicates, 171 articles were evaluated according to the title and abstract, and of these 25 articles were selected. for a complete reading of which 6 met the eligibility criteria and 19 were excluded (Appendix 3). It is noteworthy that 2 studies were included from the reference list of included articles, totaling 8 articles included [1, 3, 4, 5, 34, 35, 38, 39] in this systematic review, and none were included from the Grey Literature. The article selection process is shown in Figure 1.

Qualitative data will be explored in topics B) Additive Manufacturing Methods, C) Difference in roughness caused by different additive manufacturing methods, and D) Influence of the roughness of dental implants on osteoblastic adhesion and proliferation. To avoid biases regarding the influence of surface treatments on osteoblastic adhesion and proliferation and to consider only roughness, the focus property of this review, the results to be presented compare machined versus printed surfaces.

### 3.2 Additive manufacturing methods

Among the additive manufacturing techniques evaluated in the included studies, direct laser fabrication (DLF) is referred to in the literature as directed energy deposition, and direct metal laser sintering (DMLS), selective laser melting (SLM) and electron beam melting (EBM), which are all classified as powder bed fusion, as well as their respective printing parameters, which, when they vary, directly influence the final structure (Table 1).

**Table 1:** Additive manufacturing technique.

Author, year	Additive manufacturing technique and parameters
**Mangano et al. 2008**	DLF
	Powder Ti–6Al–4V 25–45 μm in diameter;
	Ytterbium fiber laser (EOS, GmbH Munchen, Germany);
	Scanning speed of 7 m/s; wavelength of 1054 nm;
	continuous power: 200 W;
	laser size: 0.1 mm.
**Hyzy et al. 2016**	DMLS
	Powder Ti–6Al–4V 24–45 μm in diameter;
	Ytterbium fiber laser (EOS, EmbH Munchen, Germany); scanning speed of 7 m/s;
	wavelength of 1054 nm;
	continuous power: 200 W;
	laser size: 0.1 mm.
**Ren et al. 2021**	EBM
	Powder Ti–6Al–4V 45–106 μm in diameter;
	Scanning speed of 800 mm·s^−1^;
	Power: 900 W; beam diameter of 100 μm
	layer thickness of 50 μm.
**Yu et al. 2020**	EBM
	3D printing machine (Q10 plus, Arcam, Sweden)
	Powder Ti–6Al–4V 45–106 μm in diameter;
	Scanning speed of 800 mm·s^−1^; beam diameter of 100 μm
**Mashhadi et al. 2021**	SLM
	Powder Ti grade V and 316 steel;
	Unspecified print parameters.
**Shaoki et al. 2016**	SLM
	Powder Ti IV;
	SLM® 125 HL machine (SLM Solutions GmbH, Germany);
	Scanning speed: 275 mm/s^−1^;
	Power: 100 W; layer thickness: 0.03 mm
**Suresh et al. 2021**	SLM
	Powder Ti–6Al–4V;
	EOS M290 (EOS,
	Germany);
	Dense sample:
	Scanning speed: 1200 mm/s;
	Power: 280 W;
	Layer thickness: 0.03 mm;
	Energy density: 55.56 J/mm^3^.
	Lattice-structured:
	Scanning speed: 1380 mm/s;
	Power: 252 W;
	Layer thickness: 0.03 mm;
	6.09 J/mm^2^.
**Tsukanaka et al. 2016**	SLM
	Powder CpTi <45 μm;
	EOSINT M270 SLM machine (Electro Optical Systems, Krailing, Germany);
	Scanning speed: 225 mm/s;
	Power: 117 W;
	Hatch spacing, 180 μm;
	Hatch offset, 20 μm.

In general, it is observed that the printing parameters for the DLF and DMLS techniques were similar with modification of the technique and machine. For the EBM technique, the studies used similar parameters. While for SLM there was a divergence in the parameters between the evaluated studies.

### 3.3 Difference in roughness caused by different additive manufacturing methods

According to Tables 2 and 3, the manufacturing technique influences the roughness of the printed surfaces based on the evaluated parameters Sa and Ra. It is noteworthy that the difference in roughness observed in Tables 2 and 3 must be evaluated with caution, as there was a variation in manufacturing technique, method of evaluating roughness, and roughness parameter evaluated.

**Table 2:** Roughness (Sa) per additive manufacturing technique.

Author, year	Additive manufacturing technique	Groups	Method of evaluating roughness	Results parameter Sa
**Hyzy et al. 2016**	DMLS	Ti–6Al–4V	Laser scanning microscope	Sa
	Powder Ti–6Al–4V 25–45 μm in diameter;	G1 = computer numerical control milled + polished;		G1 = 1,42 ± 0,10 μm;
	Ytterbium fiber laser (EOS, GmbH Munchen, Germany);	G2 = sintered a laser + polished;		G2 = 1,71 ± 0,05 μm;
	Scanning speed of 7 m/s; wavelength of 1054 nm;	G3 = sintered a laser + grit blasted;		G3 = 2,39 ± 0,28 μm;
	continuous power: 200 W;	G4 = sintered a laser + blasted + acid etched;		G4 = 2,94 ± 0,32 μm.
	laser size: 0.1 mm.			
**Ren et al. 2021**	EBM	Ti–6Al–4V	Laser scanning microscope	Sa
	Powder Ti–6Al–4V 45–106 μm in diameter;	G1 = EBM;		G1 = 25.51 ± 3.17 μm;
	Scanning speed of 800 mm·s^−1^;	G2 = EBM + etched in a mixed acid solution;		G2 = 16.34 ± 1.87 μm;
	Power: 900 W; beam diameter of 100 μm	G3 = EBM + anodic oxidation;		G3 = 15.62 ± 1.85 μm;
	layer thickness of 50 μm.	G4 = forged + polished.		G4 = 0.17 ± 0.04 μm.
**Yu et al. 2020**	EBM	Ti–6Al–4V	3-D measuring laser microscope	Sa
	3D printing machine (Q10 plus, Arcam, Sweden)	G1 = forged + polished;		G1 = 0.652 μm;
	Powder Ti–6Al–4V 45–106 μm in diameter;	G2 = EBM;		G2 = 13.702 μm;
	Scanning speed of 800 mm·s^−1^; beam diameter of 100 μm	G3 = EBM + soaked in a mixed solution.		G3 = 14.388 μm.

**Table 3:** Roughness (Ra) per additive manufacturing technique.

Author, year	Additive manufacturing technique	Groups	Method of evaluating roughness	Results parameter Ra
**Mangano et al. 2008**	DLF	Ti–6Al–4V	SEM	Ra
	Powder Ti–6Al–4V 25–45 μm in diameter;	G1 = DLF;		
	Ytterbium fiber laser (EOS GmbH Munchen, Germany);	G2 = smooth machined;		
	Scanning speed of 7 m/s; wavelength of 1054 nm;	G3 = smooth-machined + two grit-blasted + acid-etched Friadent Plus®;		G1 presents the highest, but the article does not express the values.
	continuous power: 200 W;	G4 = smooth-machined + Friadent DPS®.		
	laser size: 0.1 mm.			
**Mashhadi et al. 2021**	SLM	G1 = Ti grade 5 machined;	TR200	Ra
		G2 = Ti grade 2 machined;		G1 = 1.447 μm;
		G3 = 316 steel machined;		G2 = 1.253 μm;
		G4 = 304 steel machined;		G3 = 0.774 μm;
	Powder Ti grade V and 316 steel; Unspecified print parameters.	G5 = Ti grade 5 by SLM;		G4 = 0.481 μm;
		G6 = 316 steel by SLM;		G5 = 4.879 μm;
		G7 = 316 steel by SLM + machined thread.		G6 = 7.812 μm;
				G7 = 6.793 μm.
**Shaoki et al. 2016**	SLM	Ti CP grade IV	profilometer	Ra
	Powder Ti IV; SLM® 125 HL machine (SLM Solutions GmbH, Germany); Scanning speed: 275 mm/s^−1^;	G1 = SLM;		G1 = 10.65 ± 2.3 μm;
	Power: 100 W; layer thickness: 0.03 mm	G2 = machined.		G2 = 0.33 ± 0.12 μm.
**Suresh et al. 2021**	SLM	Ti–6Al–4V ELI	MR200	Ra
	Powder Ti–6Al–4V;			
	EOS M290 (EOS,			
	Germany);			
	Dense sample:			
	Scanning speed: 1200 mm/s;			
	Power: 280 W;			
	Layer thickness: 0.03 mm;	G1 = machined; G2 = dense by SLM; G3 = porous by SLM.		G3 was statistically significant in relation to G1 and G2, which did not show significant diferences, but the article does not express the values.
	Energy density: 55.56 J/mm^3^.			
	Lattice-structured:			
	Scanning speed: 1380 mm/s;			
	Power: 252 W;			
	Layer thickness: 0.03 mm;			
	6.09 J/mm^2^.			
**Tsukanaka et al. 2016**	SLM	Ti	3-D measuring laser microscope	Ra
	Powder CpTi <45 μm;	G1 = polished; G2 = SLM; G3 = SLM + alkali treatment + heat treatment.		G1 = 1.02 μm; G2 = 24.58 μm; G3 = 23.50 μm.
	EOSINT M270 SLM machine (Electro Optical Systems, Krailing, Germany);			
	Scanning speed: 225 mm/s;			
	Power: 117 W;			
	Hatch spacing, 180 μm;			
	Hatch offset, 20 μm.			

For Sa, it is observed that the EBM method presented the highest roughness when compared to DMLS. When comparing the studies by Ren et al. and Yu et al. who evaluated the EBM technique, the first one presented the highest roughness despite using the same alloy and powder granulation, scanning speed, and beam diameter, a result that can be suggested due to the variation of some unreported printing parameter.

For Ra, it is not possible to infer which DLF or SLM technique presented greater roughness due to the difference in the method of evaluating roughness between the studies. When comparing studies that used the SLM technique, the study by Tsukanaka et al. presented greater roughness, but it is noteworthy that this result should be evaluated with caution in this comparison, as there was variation in the studies for the type of alloy, printing parameters, and method of evaluating roughness that interferes in the quantitative results obtained for roughness.

### 3.4 Influence of the roughness of dental implants on osteoblastic adhesion and proliferation

Table 4 demonstrates the influence of additive manufacturing techniques on osteoblastic adhesion. In general, it is observed that when comparing machined and printed surfaces in the study by Tsukanaka et al. 2016 there was no significant difference, in those of Mangano et al. 2008 and Ren et al. 2021 the machined ones showed greater adhesion, and in those of Mashaddi et al. 2021, Shaoki et al. 2016, and Yu et al. 2020, the printed ones showed greater adherence.

**Table 4:** Influence of additive manufacturing technique on osteoblastic adhesion.

Author, year	Chemical composition and Groups	Additive Manufacturing Technique	Method to assess the roughness	Roughness results	Method to assess osteoblastic adhesion.	Cell	Osteoblastic adhesion results
Tsukanaka et al. 2016	Ti	SLM	3-D measuring laser microscope	Ra	SEM	Primary mouse osteoblasts	When comparing machined and printed surfaces there were no differences.
	G1 = polished;			G1 = 1.02 μm;			
	G2 = SLM;			G2 = 24.58 μm;			
	G3 = SLM + alkali treatment + heat treatment.			G3 = 23.50 μm.			
Mangano et al. 2008	Ti–6Al–4V	DLF	SEM	Ra	SEM	primary osteoblasts obtained by old rat calvarial parietal bone.	When comparing machined and printed surfaces, machined was better.
	G1 = DLF;			G1 presents the highest, but the article does not express the values.			
	G2 = smooth machined;						
	G3 = smooth-machined + two grit-blasted + acid-etched Friadent Plus®;						
	G4 = smooth-machined + Friadent DPS®.						
Ren et al. 2021	Ti–6Al–4V	EBM	laser scanning microscope	Sa	fluorescence microscopy and MTT	MC3T3-E1	When comparing machined and printed surfaces, machined was better.
	G1 = EBM;			G1 = 25.51 ± 3.17 μm;			
	G2 = EBM + etched in a mixed acid solution;			G2 = 16.34 ± 1.87 μm;			
	G3 = EBM + anodic oxidation;			G3 = 15.62 ± 1.85 μm;			
	G4 = forged + polished.			G4 = 0.17 ± 0.04 μm.			
Mashhadi et al. 2021	G1 = Ti grade 5 machined;	SLM	TR 200	Ra	SEM	MG-63	When comparing machined and printed surfaces, the printed was better.
	G2 = Ti grade 2 machined;			G1 = 1.447 μm;			
	G3 = 316 steel machined;			G2 = 1.253 μm;			
	G4 = 304 steel machined;			G3 = 0.774 μm;			
	G5 = Ti grade 5 by SLM;			G4 = 0.481 μm;			
	G6 = 316 steel by SLM;			G5 = 4.879 μm;			
	G7 = 316 steel by SLM + machined thread.			G6 = 7.812 μm;			
				G7 = 6.793 μm.			
Shaoki et al. 2016	Ti CP grade IV	SLM	profilometer	Ra	SEM and CCK-8	MC3T3-E1	When comparing machined and printed surfaces, the printed was better.
	G1 = SLM;			G1 = 10.65 ± 2.3 μm;			
	G2 = machined.			G2 = 0.33 ± 0.12 μm.			
Yu et al. 2020	Ti–6Al–4V	EBM	3D laser scanning microscope	Sa	confocal laser scanning microscope and MTT	MC3T3-E1	When comparing machined and printed surfaces, the printed was better.
	G1 = forged + polished;			G1 = 0.652 μm;			
	G2 = EBM;			G2 = 13.702 μm;			
	G3 = EBM + soaked in a mixed solution.			G3 = 14.388 μm.			

Table 5 demonstrates the influence of additive manufacturing techniques on osteoblastic proliferation. In general, it is observed that when comparing machined and printed surfaces in the study by Tsukanaka et al. 2016 and Ren et al. 2021 there was no significant difference, in Mashhadi et al. 2021 the machined ones showed greater proliferation and in those of Hyzy et al. 2016, Shaoki et al. 2016, Suresh et al. 2021, and Yu et al. 2020, the printed ones showed greater proliferation.

**Table 5:** Influence of additive manufacturing technique on osteoblastic proliferation.

Author, year	Chemical composition and Groups	Additive Manufacturing Technique	Method to assess the roughness	Roughness results	Method to assess osteoblastic proliferation	Cell	Osteoblastic proliferation results
Ren et al. 2021	Ti–6Al–4V	EBM	laser scanning microscope	Sa	MTT	MC3T3-E1	When comparing machined and printed surfaces there were no significant differences.
	G1 = EBM;			G1 = 25.51 ± 3.17 μm;			
	G2 = EBM + etched in a mixed acid solution;			G2 = 16.34 ± 1.87 μm;			
	G3 = EBM + anodic oxidation;			G3 = 15.62 ± 1.85 μm;			
	G4 = forged + polished.			G4 = 0.17 ± 0.04 μm.			
Tsukanaka et al. 2016	Ti	SLM	3-D measuring laser microscope	Ra	XTT	Primary mouse osteoblasts	When comparing machined and printed surfaces there were no significant differences.
	G1 = polished;			G1 = 1.02 μm;			
	G2 = SLM;			G2 = 24.58 μm;			
	G3 = SLM + alkali treatment + heat treatment.			G3 = 23.50 μm.			
Mashhadi et al. 2021	G1 = Ti grade 5 machined;	SLM	TR 200	Ra	SEM	MG63	When comparing machined and printed surfaces, the machined ones were better.
	G2 = Ti grade 2 machined;			G1 = 1.447 μm;			
	G3 = 316 steel machined;			G2 = 1.253 μm;			
	G4 = 304 steel machined;			G3 = 0.774 μm;			
	G5 = Ti grade 5 by SLM;			G4 = 0.481 μm;			
	G6 = 316 steel by SLM;			G5 = 4.879 μm;			
	G7 = 316 steel by SLM + machined thread.			G6 = 7.812 μm;			
				G7 = 6.793 μm.			
Hyzy et al. 2016	Ti–6Al–4V	DMLS	laser scanning microscope	Sa	mRNA analysis and Secreted factors analysis (ALP, OCN, OPG, FGF2,BMP2, and VEGF.)	MG63	When comparing machined and printed surfaces, the printed was better.
	G1 = computer numerical control milled + polished;			G1 = 1,42 ± 0,10 μm;			
	G2 = sintered a laser + polished;			G2 = 1,71 ± 0,05 μm;			
	G3 = sintered a laser + grit blasted;			G3 = 2,39 ± 0,28 μm;			
	G4 = sintered a laser + blasted + acid etched;			G4 = 2,94 ± 0,32 μm.			
Shaoki et al. 2016	Ti CP grade IV	SLM	profilometer	Ra	CCK-8	MC3T3-E1	When comparing machined and printed surfaces, the printed was better.
	G1 = SLM;			G1 = 10.65 ± 2.3 μm;			
	G2 = machined.			G2 = 0.33 ± 0.12 μm.			
Suresh et al. 2021	Ti–6Al–4V ELI	SLM	MR200	Ra	CTG	MC3T3-E1	When comparing machined and printed surfaces, the printed was better.
	G1 = machined;			G3 was statistically significant in relation to G1 and G2, which did not show significant differences.			
	G2 = dense by SLM;						
	G3 = porous by SLM.						
Yu et al. 2020	Ti–6Al–4V	EBM	3D laser scanning microscope	Sa	MTT	MC3T3-E1	When comparing machined and printed surfaces, the printed was better.
	G1 = forged + polished;			G1 = 0.652 μm;			
	G2 = EBM;			G2 = 13.702 μm;			
	G3 = EBM + soaked in a mixed solution.			G3 = 14.388 μm.			

### 3.5 Meta-analysis

The studies included in this systematic review showed heterogeneity in terms of chemical composition, additive manufacturing technique, the method to evaluate roughness, evaluated roughness parameter, and method to evaluate adhesion and/or osteoblastic and cell proliferation, factors which made quantitative meta-analysis impossible.

### 3.6 Risk of bias

**Figure 2:**
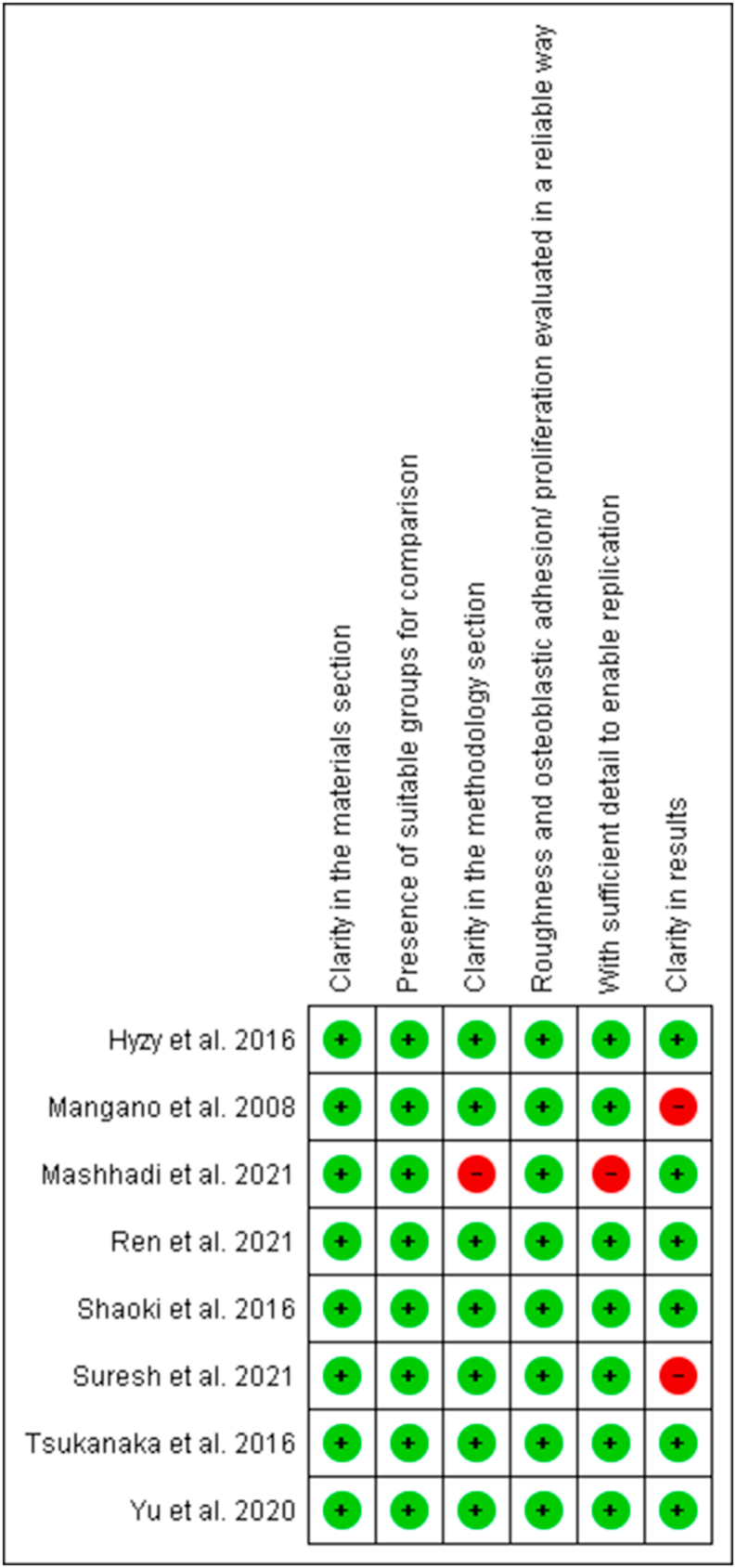
Assessment of the risk of bias of included studies.

When analyzing the risk of bias, 7 studies had a low risk of bias and 1 had a moderate risk of bias. The risk of bias in the studies by Mangano et al. 2008 and Suresh et al. 2021 was increased in the “clarity in the results” factor because they did not express the roughness value of the samples, while Mashhadi et al. 2021 was increased to “clarity in the methodology section” for not specifying the printing parameters used, which makes its replication unfeasible, increasing the risk for the factor “with sufficient detail to enable replication” (Figure 2).

## 4 Discussion

Osteoblastic adhesion and proliferation on the implant surface during contact osteogenesis are essential for the phenomenon of osseointegration being influenced by roughness variations, a property strongly altered in implants printed by additive manufacturing. The critical and careful analysis of the 8 in vitro articles included in this systematic review, heterogeneous in terms of chemical composition, additive manufacturing technique, the method to evaluate roughness, evaluated roughness parameter, method to evaluate adhesion and/or osteoblastic proliferation, and cell allowed to partially respond to the question and will be addressed in sections 1) Additive Manufacturing Methods, 2) Difference in roughness caused by different additive manufacturing methods, 3) Influence of the roughness of dental implants on osteoblastic adhesion and proliferation, and 4) Final considerations.

### 4.1 Additive manufacturing methods

The articles included in this systematic review presented the additive manufacturing processes directed energy deposition (DED) by the DLF [4] and powder bed fusion (PBF) by DMLS [3], EBM [1, 5], and SLM [34, 35, 38, 39]. The review by Svetlizky et al. [40] when comparing the two processes it is inferred that the PBF process ensures a better resolution of the parts, while the DED ones are repairable. Although both processes use electrons or laser beams as sources of energy, their deposition differs, since in PBF the metallic powder is deposited on the printing platform to be melted, whereas in DED the powder is deposited melted through a nozzle on the printing platform [40].

Among these two processes, PBF is the most used and its energy source can be through an electron beam such as EBM or a laser beam such as DMLS and SLM [41, 42, 43, 44]. The EBM technique emits electrons from a tungsten filament of mode controlled by two magnetic fields that are the focusing coil that determines the diameter of the beam and the deflecting coil that deflects the electron beam in the dust layer [43]. The DMLS and SLM techniques emit a laser beam in the powder layer and the main difference is the temperature to which the powder is subjected, which is higher in SLM, so it is preferable to use pure metals as raw materials and DMLS metallic alloys [40, 42].

The qualitative analysis of the EBM, DLF, DMLS, and SLM techniques and the impression parameters of the studies included [1, 3, 4, 5, 34, 35, 38, 39] in this systematic review allowed us to infer that all produced a biocompatible roughness for adhesion and osteoblastic proliferation. In light of the fact that the roughness of the structure produced is influenced by printing parameters such as platform orientation, energy flow, powder granulation, and layer thickness during construction, the authors of this review encourage further research that identifies the best technique and impression parameter for osteoblast adhesion and proliferation by accurately comparing these techniques through methodologies and similar production parameters.

It is noteworthy that surface treatments and polishing are proposed on the printed surfaces to regularize the surface and remove loosely adhered spherical particles, which can lead to peri-implant inflammation and significantly affect osseointegration [1, 3, 4, 5, 12, 19, 21, 35, 45, 46, 47, 48, 49, 50, 51]. Among them, biomimetic treatments and the hierarchical structure of bone tissue are stimulated by creating a harmonic environment similar to the natural one for the events of osteogenesis [1, 5, 35, 45, 52].

Studies show that micro, macro, and nanoscale roughness favors osteoblastic differentiation more than just one [5, 53, 54]. As demonstrated in the included studies by Mangano et al. [4], and Ren et al. [5], Shimizu et al. [45], Tsukanaka et al.. [35], and Yu et al. [1] that the applied surface treatments (Appendix 2) provided a micro and nanometric topography with greater bioactivity that allowed greater osteoblastic adhesion [1, 4, 5, 35, 45] and proliferation [1, 5, 35].

### 4.2 Differences in roughness caused by different additive manufacturing methods

The evaluated studies showed divergence regarding the roughness evaluation method, evaluated roughness parameter, Ra and Sa, and printing parameters, such as powder granulation, printing speed, and power when using the same technique, which prevented a comparison need between the techniques. When analyzing the Sa parameter, it can be inferred that the EBM technique provides rougher surfaces than the DMLS, and, regarding the Ra parameter, the absence of numerical data for the roughness produced by the DLF technique in the study by Mangano et al. made it impossible to compare it with studies that evaluated SLM [34, 35, 38, 39].

In cellular terms, the literature is still very controversial about the best roughness value for osteoblastic adhesion and proliferation, as reiterated by the studies included in this review [1, 3, 4, 5, 34, 35, 38, 39]. As the difference in roughness caused by the different additive manufacturing methods evaluated is an important parameter to be considered, the authors of this review can infer that all the values found were biocompatible for osteoblastic adhesion and proliferation and it is not possible to determine the best roughness and technique due to the heterogeneity of the studies. However, as the increase in this favors the increase in the available surface area in vivo, it may favor the mechanical imbrication [34, 35, 55, 56, 57, 58], while, in cellular terms, this should be correlated with the morphology of the peaks and valleys as well as the hydrophilicity and electrostatic condition of the surface that interact synergistically for osseointegration [59].

### 4.3 Influence of the roughness of dental implants on osteoblastic adhesion and proliferation

After insertion of an implant, the first phenomenon to occur is their wetting by the blood and consequent adsorption of blood plasma proteins, fibronectin, and vibronectin, depending on their wettability, at the same time bone morphogenetic proteins (BMPs) are activated by trauma surgery and induce the differentiation of mesenchymal stem cells linked to proteins anchored in the biomaterial into osteoblasts, after the formation of immature bone tissue, bone remodeling, synchronous action of osteoblasts and osteoclasts, will dictate osseointegration [60, 61, 62, 63]. Since osteogenesis is influenced by the topographic characteristics of the surface, which modulate bone cell adhesion and proliferation behavior by altering signaling pathways for mineralization of the extracellular matrix, as well as the fact that there is still no consensus in the literature about how roughness impacts adhesion and osteoblast proliferation, we will discuss how it impacts machined and printed surfaces.

The mechanisms by which different topographies induce focal adhesion of osteoblasts are still unknown in the literature, the preference for Ra surfaces between 3–5 μm than smooth surfaces of Ra <1 μm is elucidated because they present higher surface-free energy for clot adhesion, proteins, and growth factors that are precursors of osteoblastos [5, 33, 34, 64]. However, in this review Mangano et al. [4], Ren et al. [5], and Tsukanaka et al. [35]differ from the literature by demonstrating greater [4, 5] and absence of differences [35] for adhesion between machined surfaces and those printed by DLF [4], EBM [5], and SLM [35], these results are attributed to the morphology of the peaks of the printed surfaces do not favor osteoblastic adhesion.

Thus, the authors of this review emphasize that the morphology of the peaks interferes more with the osteoblastic adhesion than the quantitative value of the roughness since the osteoblastic cells have a greater tendency to adhere to them because they present greater surface tension than the valleys so that these are rounded and not sharpened, reduced adhesion will occur.

The literature is still unclear concerning surfaces of Ra>5 μm, such as those obtained by additive manufacturing, which present Ra around 25μm and biomimic trabecular bone, thus suggesting that they can increase bone neoformation [5, 29, 32, 34, 35, 54]. Mashaddi et al. [38], Shaoki et al. [34], and Yu et al. [1] corroborate by demonstrating that the surface obtained by SLM [34, 38] and EBM [1] showed greater osteoblastic adhesion than the machined ones, possibly due to biomimetic topography.

The authors of this review suggest that printed surfaces were associated with greater osteoblastic adhesion because they biomimic the bone tissue, they modulate the signaling pathways of integrins, α1, β1, and β3, proteins that regulate osteoblastic cell adhesion in the biomaterial, to favor the formation of focal adhesions and the extension of cellular filopodia [56, 65, 66].

There is also no consensus in the literature for cell proliferation, while authors [61, 67, 68, 69] infer that rough surfaces promote a more osteogenic phenotype that favors the expression of growth factors by bone cells such as alkaline phosphatase (ALP), osteocalcin (OCN), osteoprotegerin (OPG), and bone morphogenetic protein (BMP2). Groessner-Schreiber et al. [70] disagree and infer that the increase in roughness favors adhesion, but reduces the rate of cell proliferation.

Of the 7 articles included [1, 3, 5, 34, 35, 39, 71] in this review that compared osteoblastic cell proliferation between machined and printed surfaces, it was observed that most Hyzy et al. 2016 [3], Shaoki et al. 2016 [34], Suresh et al. 2021 [39], and Yu et al. 2020 [1] reported increased proliferation on printed surfaces. According to the authors of this review, this fact can be attributed to the greater surface area provided by the porosity and consequent roughness of the printed surfaces for the anchoring of proteins and bone cells.

### 4.4 Final considerations

The question that motivated this systematic review can be partially answered, because although we observe the influence of roughness when comparing machined and printed surfaces, of different roughness, heterogeneous data regarding chemical composition, additive manufacturing technique, the method to evaluate roughness, evaluated roughness, the method to evaluate adhesion and/or osteoblastic proliferation and cell the divergent results do not allow to infer how roughness interferes with osteoblastic adhesion and proliferation, being necessary more studies at the biomolecular level to understand this process.

## Declarations

### Author contribution statement

Juliana Dias Corpa Tardellia and Andréa Cândido dos Reisa: Conceived and designed the experiments; Performed the experiments; Analyzed and interpreted the data; Contributed reagents, materials, analysis tools or data; Wrote the paper.

Ana Carolina Duarte Firminoa and Izabela Ferreiraa: Conceived and designed the experiments.

### Funding statement

Juliana Dias Corpa Tardelli was supported by FAPESP [2020/05272-2].

### Data availability statement

This is a systematic review, so the datas were detailed in the manuscript.

### Declaration of interest’s statement

'The authors declare no competing interests.

### Additional information

Supplementary content related to this article has been published online at [URL].
