# Supplementary material for: Influence of the roughness of dental implants obtained by additive manufacturing on osteoblastic adhesion and proliferation: A systematic review
Source: Heliyon. 2022 Dec 25;8(12):e12505. doi: 10.1016/j.heliyon.2022.e12505 (PMC9834751; doi:10.1016/j.heliyon.2022.e12505)
Supplement: Appendix 1 [file mmc1.docx]

**Appendix 1** - Database search strategy.

| **Database** | **Search** | **Found** |
| --- | --- | --- |
| **EMBASE**  October  03^rd,^ 2022 | (‘dental implant’ OR ‘additive manufacturing’ OR ‘3D printing’) AND roughness AND (machined OR forged OR ‘subtractive manufacturing’) AND (‘cell viability’ OR ‘cell adhesion’ OR ‘osteoblast’) | 36 |
| **PubMed**  October  03^rd,^ 2022 | (“dental implant” OR “additive manufacturing” OR “3D printing”) AND roughness AND (machined OR forged OR “subtractive manufacturing”) AND (“cell viability” OR “cell adhesion” OR “osteoblast”) | 29 |
| **Scopus**  October  03^rd,^ 2022 | (“dental implant” OR “additive manufacturing” OR “3D printing”) AND roughness AND (machined OR forged OR “subtractive manufacturing”) AND (“cell viability” OR “cell adhesion” OR “osteoblast”) | 58 |
| **Science Direct**  October  03^rd,^ 2022 | “dental implant” AND (“additive manufacturing” OR “3D printing”) AND roughness (machined OR “subtractive manufacturing”) AND (“cell viability” OR “cell adhesion” OR “osteoblast”) | 100 |
| **Grey Literature** | | |
| **Google Scholar**  October  03^rd,^ 2022 | “dental implant” AND (“additive manufacturing” OR “3D printing”) AND roughness (machined OR “subtractive manufacturing”) AND (“cell viability” OR “cell adhesion” OR “osteoblast”) | 100 |
| **ProQuest**  October  03^rd,^ 2022 | “dental implant” AND (“additive manufacturing” OR “3D printing”) AND roughness (machined OR “subtractive manufacturing”) AND (“cell viability” OR “cell adhesion” OR “osteoblast”) | 100 |
