# Supplementary material for: Influence of the roughness of dental implants obtained by additive manufacturing on osteoblastic adhesion and proliferation: A systematic review
Source: Heliyon. 2022 Dec 25;8(12):e12505. doi: 10.1016/j.heliyon.2022.e12505 (PMC9834751; doi:10.1016/j.heliyon.2022.e12505)
Supplement: Appendix 2 [file mmc2.docx]

**Appendix 2.** Characteristics of included studies.

| Author, year | Chemical composition and Groups | Additive Manufacturing Technique | Method to assess the roughness | Roughness results | Method to assess osteoblastic adhesion and/or proliferation |  |  | Cell | Osteoblastic adhesion and/or proliferation results |  |
| --- | --- | --- | --- | --- | --- | --- | --- | --- | --- | --- |
| Hyzy et al. 2016 | Ti-6Al-4V  G1=computer numerical control milled + polished;  G2=sintered a laser + polished;  G3=sintered a laser + grit blasted;  G4=sintered a laser + blasted + acid etched; | DMLS | laser scanning microscope | Sa  G1=1,42 ± 0,10 μm;  G2= 1,71 ± 0,05 μm;  G3= 2,39 ± 0,28 μm;  G4= 2,94 ± 0,32 μm. | mRNA analysis and Secreted factors analysis(ALP, OCN, OPG, FGF2,BMP2, and VEGF.) |  |  | MG63 | DNA was higher on printed than machined surfaces. While the secreted factors were significantly higher in the superficially treated groups. |  |
| Mangano et al. 2008 | Ti-6Al-4V  G1=DLF;  G2=smooth machined;  G3=smooth-machined + two grit-blasted + acid-etched Friadent Plus®;  G4=smooth-machined + Friadent DPS®. | DLF | SEM | Ra  G1 presents the highest, but the article does not express the values. | SEM |  |  | primary osteoblasts obtained by old rat calvarial parietal bone. | Higher cell adhesion in G2 and G4 and lower in G1 and G3. |  |
| Mashhadi et al. 2021 | G1=Ti grade 5 machined;  G2=Ti grade 2 machined;  G3=316 steel machined;  G4=304 steel machined;  G5=Ti grade 5 by SLM;  G6=316 steel by SLM;  G7=316 steel by SLM + machined thread. | SLM | TR 200 | Ra  G1=1.447μm;  G2=1.253μm;  G3=0.774μm;  G4=0.481μm;  G5=4.879μm;  G6=7.812μm;  G7=6.793μm. | SEM |  |  | MG-63 | As the roughness increased, the cell adhesion increased, however, there was a decrease in cell proliferation, expansion, elongation, and migration. |  |
| Ren et al. 2021 | Ti-6Al-4V  G1=EBM;  G2=EBM + etched in a mixed acid solution;  G3=EBM + anodic oxidation;  G4= forged + polished. | EBM | laser scanning microscope | Sa  G1=25.51±3.17μm;  G2=16.34±1.87μm;  G3=15.62±1.85μm;  G4=0.17±0.04μm. | fluorescence microscopy and MTT |  |  | MC3T3-E1 | For adhesion, G1 promoted the lowest. For proliferation, G2 and G3 were higher when compared to G1 and G4. |  |
| Shaoki et al. 2016 | Ti CP grade IV  G1=SLM;  G2=machined. | SLM | profilometer | Ra  G1=10.65±2.3μm;  G2=0.33±0.12μm. | SEM and CCK-8 |  |  | MC3T3-E1 | Greater cell adhesion and proliferation in G1. |  |
| Suresh et al. 2021 | Ti-6Al-4V ELI  G1=machined;  G2=dense by SLM;  G3=porous by SLM. | SLM | MR200 | Ra  G3 was statistically significant in relation to G1 and G2, which did not show significant differences. | CTG |  |  | MC3T3-E1 | Greater proliferation in G3 followed by G2 and G1. |  |
| Tsukanaka et al. 2016 | Ti  G1=polished;  G2=SLM;  G3=SLM + alkali treatment+ heat treatment. | SLM | 3-D  measuring laser microscope | Ra  G1=1.02 μm;  G2=24.58 μm;  G3=23.50 μm. | SEM and XTT |  |  | Primary mouse osteoblasts | G3 showed greater adherence and viability due to the bioactive treatment. G2 showed no difference in adherence compared to G1. |  |
| Yu et al. 2020 | Ti-6Al-4V  G1=forged + polished;  G2=EBM;  G3=EBM + soaked in a mixed solution. | EBM | 3D laser scanning microscope | Sa  G1=0.652μm;  G2=13.702μm;  G3=14.388 μm. | confocal laser scanning microscope and MTT |  |  | MC3T3-E1 | G3 showed the highest cell adhesion and viability possibly due to micro/nanostructure and G1 the lowest. |  |

ALP, Alkaline phosphatase specific activity; BMP2, bone morphogenetic protein 2; CNC-M, disks computer numerical control milled polished; CTG, CellTiter-Glo® 3D Cell Viability Assay; DMLS, Direct metal laser sintering; FGF2, fibroblast growth factor 2; LST-B, disks sintered a laser grit blasted; LST-BE, disks sintered a laser blasted and acid etched; LST-M, disks sintered a laser polished; OCN, osteocalcin; OPG, osteoprotegerin; VEGF, vascular endothelial growth factor A.
