# Supplementary material for: Influence of the roughness of dental implants obtained by additive manufacturing on osteoblastic adhesion and proliferation: A systematic review
Source: Heliyon. 2022 Dec 25;8(12):e12505. doi: 10.1016/j.heliyon.2022.e12505 (PMC9834751; doi:10.1016/j.heliyon.2022.e12505)
Supplement: Appendix 3 [file mmc3.docx]

**Appendix 3.** Excluded articles and reasons for exclusion.

| **Author, year** | **Reason for exclusion** |
| --- | --- |
| Blatt et al., 2018 | 1 |
| Y.-D. Cho et al., 2016 | 1 |
| Y. D. Cho et al., 2021 | 1 |
| Ciliveri & Bandyopadhyay, 2022 | 2 |
| Colombo et al., 2012 | 1 |
| Dong et al., 2020 | 2 |
| Fojt et al., 2018 | 2 |
| Gupta et al., 2021 | 3 |
| Hara et al., 2012 | 1 |
| Kim et al., 2020 | 4 |
| Li et al., 2019 | 5 |
| Luo et al., 2021 | 2 |
| Ogura et al., 2022 | 2 |
| Shimizu et al., 2020 | 2 |
| Wally et al., 2019 | 2 |
| Wang et al., 2016 | 2 |
| Wang et al., 2021 | 6 |
| Weinmann et al., 2018 | 7 |
| Xu et al., 2018 | 1 |

1. Did not evaluate surfaces obtained by additive manufacturing (n=6); 2) Did not evaluate surfaces obtained by machining (n=8); 3) bone plates (n=1); 4) Compared machined surface with obtained by additive manufacturing treated superficially (n=1); 5) scaffold (n=1); 6) Did not mention additive manufacturing technique (n=1);7) orthopedic implant (n=1).

**REFERENCES**

[1] S. Blatt, A.M. Pabst, E. Schiegnitz, M. Hosang, T. Ziebart, C. Walter, B. Al-Nawas, M.O. Klein, Early cell response of osteogenic cells on differently modified implant surfaces: Sequences of cell proliferation, adherence and differentiation, J. Cranio-Maxillofacial Surg. 46 (2018) 453–460. https://doi.org/10.1016/j.jcms.2017.12.021.

[2] Y.-D. Cho, S.-J. Kim, H.-S. Bae, W.-J. Yoon, K.-H. Kim, H.-M. Ryoo, Y.-J. Seol, Y.-M. Lee, I.-C. Rhyu, Y. Ku, Biomimetic Approach to Stimulate Osteogenesis on Titanium Implant Surfaces Using Fibronectin Derived Oligopeptide, Curr. Pharm. Des. 22 (2016) 4729–4735. https://doi.org/10.2174/1381612822666160203143053.

[3] Y.D. Cho, W.J. Kim, S. Kim, Y. Ku, H.M. Ryoo, Surface topography of titanium affects their osteogenic potential through DNA methylation, Int. J. Mol. Sci. 22 (2021) 1–11. https://doi.org/10.3390/ijms22052406.

[4] S. Ciliveri, A. Bandyopadhyay, Influence of strut-size and cell-size variations on porous Ti6Al4V structures for load-bearing implants, J. Mech. Behav. Biomed. Mater. 126 (2022) 105023. https://doi.org/10.1016/j.jmbbm.2021.105023.

[5] J.S. Colombo, A. Carley, G.J.P. Fleming, S.J. Crean, A.J. Sloan, R.J. Waddington, Osteogenic potential of bone marrow stromal cells on smooth, roughened, and tricalcium phosphate-modified titanium alloy surfaces., Int. J. Oral Maxillofac. Implants. 27 (2012) 1029–1042.

[6] Y.P. Dong, J.C. Tang, D.W. Wang, N. Wang, Z.D. He, J. Li, D.P. Zhao, M. Yan, Additive manufacturing of pure Ti with superior mechanical performance, low cost, and biocompatibility for potential replacement of Ti-6Al-4V, Mater. Des. 196 (2020). https://doi.org/10.1016/j.matdes.2020.109142.

[7] J. Fojt, M. Fousova, E. Jablonska, L. Joska, V. Hybasek, E. Pruchova, D. Vojtech, T. Ruml, Corrosion behaviour and cell interaction of Ti-6Al-4V alloy prepared by two techniques of 3D printing, Mater. Sci. Eng. C. 93 (2018) 911–920. https://doi.org/10.1016/j.msec.2018.08.066.

[8] S.K. Gupta, N. Shahidsha, S. Bahl, D. Kedaria, S. Singamneni, P.K.D.V. Yarlagadda, S. Suwas, K. Chatterjee, Enhanced biomechanical performance of additively manufactured Ti-6Al-4V bone plates, J. Mech. Behav. Biomed. Mater. 119 (2021) 104552. https://doi.org/10.1016/j.jmbbm.2021.104552.

[9] T. Hara, K. Matsuoka, K. Matsuzaka, M. Yoshinari, T. Inoue, Effect of surface roughness of titanium dental implant placed under periosteum on gene expression of bone morphogenic markers in rat., Bull. Tokyo Dent. Coll. 53 (2012) 45–50. https://doi.org/10.2209/tdcpublication.53.45.

[10] J.H. Kim, M.Y. Kim, J.C. Knowles, S. Choi, H. Kang, S. hyun Park, S.M. Park, H.W. Kim, J.T. Park, J.H. Lee, H.H. Lee, Mechanophysical and biological properties of a 3D-printed titanium alloy for dental applications, Dent. Mater. 36 (2020) 945–958. https://doi.org/10.1016/j.dental.2020.04.027.

[11] J. Li, Z. Li, Y. Shi, H. Wang, R. Li, J. Tu, G. Jin, In vitro and in vivo comparisons of the porous Ti6Al4V alloys fabricated by the selective laser melting technique and a new sintering technique, J. Mech. Behav. Biomed. Mater. 91 (2019) 149–158. https://doi.org/10.1016/j.jmbbm.2018.12.007.

[12] X. Luo, C. Yang, R.Y. Li, H. Wang, H.Z. Lu, T. Song, H.W. Ma, D.D. Li, A. Gebert, Y.Y. Li, Effect of silicon content on the microstructure evolution, mechanical properties, and biocompatibility of β-type TiNbZrTa alloys fabricated by laser powder bed fusion, Mater. Sci. Eng. C. 133 (2021). https://doi.org/10.1016/j.msec.2021.112625.

[13] N. Ogura, M.B. Berger, P. Srivas, S. Hwang, J. Li, D.J. Cohen, Z. Schwartz, B.D. Boyan, K.H. Sandhage, Tailoring of TiAl6V4 Surface Nanostructure for Enhanced In Vitro Osteoblast Response via Gas/Solid (Non-Line-of-Sight) Oxidation/Reduction Reactions, Biomimetics. 7 (2022) 117. https://doi.org/10.3390/biomimetics7030117.

[14] Y. Shimizu, S. Fujibayashi, S. Yamaguchi, S. Mori, H. Kitagaki, T. Shimizu, Y. Okuzu, K. Masamoto, K. Goto, B. Otsuki, T. Kawai, K. Morizane, T. Kawata, S. Matsuda, Bioactive effects of strontium loading on micro/nano surface Ti6Al4V components fabricated by selective laser melting, Mater. Sci. Eng. C. 109 (2020) 110519. https://doi.org/10.1016/j.msec.2019.110519.

[15] Z.J. Wally, A.M. Haque, A. Feteira, F. Claeyssens, R. Goodall, G.C. Reilly, Selective laser melting processed Ti6Al4V lattices with graded porosities for dental applications, J. Mech. Behav. Biomed. Mater. 90 (2019) 20–29. https://doi.org/10.1016/j.jmbbm.2018.08.047.

[16] M. Wang, Y. Wu, S. Lu, T. Chen, Y. Zhao, H. Chen, Z. Tang, Fabrication and characterization of selective laser melting printed Ti–6Al–4V alloys subjected to heat treatment for customized implants design, Prog. Nat. Sci. Mater. Int. 26 (2016) 671–677. https://doi.org/10.1016/j.pnsc.2016.12.006.

[17] H. Wang, J. Liu, C. Wang, S.G. Shen, X. Wang, K. Lin, The synergistic effect of 3D-printed microscale roughness surface and nanoscale feature on enhancing osteogenic differentiation and rapid osseointegration, J. Mater. Sci. Technol. 63 (2021) 18–26. https://doi.org/10.1016/j.jmst.2019.12.030.

[18] M. Weinmann, C. Schnitter, M. Stenzel, J. Markhoff, C. Schulze, R. Bader, Development of bio-compatible refractory Ti/Nb(/Ta) alloys for application in patient-specific orthopaedic implants, Int. J. Refract. Met. Hard Mater. 75 (2018) 126–136. https://doi.org/10.1016/j.ijrmhm.2018.03.018.

[19] W. Xu, X. Lu, L.N. Wang, Z.M. Shi, S.M. Lv, M. Qian, X.H. Qu, Mechanical properties, in vitro corrosion resistance and biocompatibility of metal injection molded Ti-12Mo alloy for dental applications, J. Mech. Behav. Biomed. Mater. 88 (2018) 534–547. https://doi.org/10.1016/j.jmbbm.2018.08.038.
